# Supplementary material for: End Sequence Analysis Toolkit (ESAT) expands the extractable information from single-cell RNA-seq data
Source: Genome Res. 2016 Oct;26(10):1397–410. doi: 10.1101/gr.207902.116 (PMC5052061; doi:10.1101/gr.207902.116)
Supplement: Supplemental Material [file supp_gr.207902.116_Supp_Materials.tar.gz › ESAT-esat_v0.1/doc/html/classbroad_1_1core_1_1annotation_1_1_maximum_contiguous_subsequence.html]

ESAT: broad.core.annotation.MaximumContiguousSubsequence Class Reference


ESAT


- Main Page
- Related Pages
- Packages
- Classes
- Files

- Class List
- Class Index
- Class Hierarchy
- Class Members

All Classes Namespaces Files Functions Variables Enumerator Pages

- broad
- core
- annotation
- MaximumContiguousSubsequence

Static Public Member Functions |
List of all members

broad.core.annotation.MaximumContiguousSubsequence Class Reference

|  |  |
| --- | --- |
| Static Public Member Functions | |
| static int[] | maxSubSum3 (int[] a) |
|  | |
| static double[] | maxSubSum3 (double[] a) |
|  | |
| static int | contiguousStartSubSequenceOverMin (double[] a, double min) |
|  | |
| static int | contiguousEndSubSequenceOverMin (double[] a, double min) |
|  | |
| static void | main (String[] args) |
|  | |

## Member Function Documentation

|  |  |  |  |
| --- | --- | --- | --- |
| static int broad.core.annotation.MaximumContiguousSubsequence.contiguousEndSubSequenceOverMin | ( | double[] | *a*, |
|  |  | double | *min* |
|  | ) |  |  |

static

|  |  |  |  |
| --- | --- | --- | --- |
| static int broad.core.annotation.MaximumContiguousSubsequence.contiguousStartSubSequenceOverMin | ( | double[] | *a*, |
|  |  | double | *min* |
|  | ) |  |  |

static

|  |  |  |  |  |  |
| --- | --- | --- | --- | --- | --- |
| static void broad.core.annotation.MaximumContiguousSubsequence.main | ( | Stringargs | [] | ) |  |

static

Here is the call graph for this function:

|  |  |  |  |  |  |
| --- | --- | --- | --- | --- | --- |
| static int [] broad.core.annotation.MaximumContiguousSubsequence.maxSubSum3 | ( | int[] | *a* | ) |  |

static

Linear-time maximum contiguous subsequence sum algorithm. seqStart and seqEnd represent the actual best sequence.

Here is the caller graph for this function:

|  |  |  |  |  |  |
| --- | --- | --- | --- | --- | --- |
| static double [] broad.core.annotation.MaximumContiguousSubsequence.maxSubSum3 | ( | double[] | *a* | ) |  |

static

---

The documentation for this class was generated from the following file:

- java/broad/core/annotation/MaximumContiguousSubsequence.java


---

Generated on Fri Aug 15 2014 15:10:51 for ESAT by  

 1.8.7
